# Gene expression of the liver of vaccination-protected mice in response to early patent infections of *Plasmodium chabaudi* blood-stage malaria

**DOI:** 10.1186/s12936-018-2366-6

**Published:** 2018-05-29

**Authors:** Saleh Al-Quraishy, Mohamed A. Dkhil, E. M. Al-Shaebi, Abdel-Azeem S. Abdel-Baki, Marcos J. Araúzo-Bravo, Denis Delic, Frank Wunderlich

**Affiliations:** 1 Department of Zoology, College of Science, King Saud University, P.O. Box: 2455, Riyadh, 11451 Saudi Arabia; 2 Department of Zoology and Entomology, Faculty of Science, Helwan University, Cairo, Egypt; 3 Department of Zoology, Faculty of Science, Beni-Suef University, Beni-Suef, Egypt; 4 Group of Computational Biology and Systems Biomedicine, Biodonostia Health Research Institute, San Sebastián, Spain; 5 IKERBASQUE, Basque Foundation for Science, Bilbao, Spain; 6 Boehringer-Ingelheim Pharma, Biberach, Germany; 7 Department of Biology, Heinrich-Heine-University, Düsseldorf, Germany

**Keywords:** *Plasmodium chabaudi*, Blood-stage malaria, Liver, Gene expression, Vaccination, Extramedullary erythropoiesis, Natural cytotoxicity

## Abstract

**Background:**

The role of the liver for survival of blood-stage malaria is only poorly understood. In experimental blood-stage malaria with *Plasmodium chabaudi*, protective vaccination induces healing and, thus, survival of otherwise lethal infections. This model is appropriate to study the role of the liver in vaccination-induced survival of blood-stage malaria.

**Methods:**

Female Balb/c mice were vaccinated with a non-infectious vaccine consisting of plasma membranes isolated in the form of erythrocyte ghosts from *P. chabaudi*-infected erythrocytes at week 3 and week 1 before infection with *P. chabaudi* blood-stage malaria. Gene expression microarrays and quantitative real-time PCR were used to investigate the response of the liver, in terms of expression of mRNA and long intergenic non-coding (linc)RNA, to vaccination-induced healing infections and lethal *P. chabaudi* malaria at early patency on day 4 post infection, when parasitized erythrocytes begin to appear in peripheral blood.

**Results:**

In vaccination-induced healing infections, 23 genes were identified to be induced in the liver by > tenfold at p < 0.01. More than one-third were genes known to be involved in erythropoiesis, such as *Kel*, *Rhag*, *Ahsp*, *Ermap*, *Slc4a1*, *Cldn13 Gata1*, and *Gfi1b*. Another group of > tenfold expressed genes include genes involved in natural cytotoxicity, such as those encoding killer cell lectin-like receptors *Klrb1a*, *Klrc3*, *Klrd1*, the natural cytotoxicity-triggering receptor 1 *Ncr1*, as well as the granzyme B encoding *Gzmb*. Additionally, a series of genes involved in the control of cell cycle and mitosis were identified: *Ccnb1*, *Cdc25c*, *Ckap2l* were expressed > tenfold only in vaccination-protected mice, and the expression of 22 genes was at least 100% higher in vaccination-protected mice than in non-vaccinated mice. Furthermore, distinct lincRNA species were changed by > threefold in livers of vaccination-protected mice, whereas lethal malaria induced different lincRNAs.

**Conclusion:**

The present data suggest that protective vaccination accelerates the malaria-induced occurrence of extramedullary erythropoiesis, generation of liver-resident cytotoxic cells, and regeneration from malaria-induced injury in the liver at early patency, which may be critical for final survival of otherwise lethal blood-stage malaria of *P. chabaudi*.

**Electronic supplementary material:**

The online version of this article (10.1186/s12936-018-2366-6) contains supplementary material, which is available to authorized users.

## Background

Malaria is still one of the most life-threatening infectious diseases in tropical countries. The World Health Organization (WHO) had estimated about 212 million new cases and about 429,000 deaths globally in 2015, with about 70% of total deaths occurring in children aged under 5 years [1]. An effective anti-malarial vaccine is not yet commercially available [2–4].

Morbidity and mortality from malaria are caused by the blood stages of the malaria-causing agent, parasitic protozoans of the genus *Plasmodium*, which develop within host erythrocytes. The spleen with its inherent mechanism to remove senescent and other aberrant erythrocytes from circulation is currently thought to be the exclusive effector organ to eliminate *Plasmodium*-parasitized erythrocytes from circulation [5]. However, the liver is also equipped with effective mechanisms for removing aberrant erythrocytes including *Plasmodium*-infected erythrocytes [6–11]. The liver with its intrinsic immune system is therefore increasingly, though still hesitantly, recognized as an effector organ against blood-stage malaria [10]. *Plasmodium chabaudi* infection in mice is an appropriate model to study the effector functions of the liver against blood-stage malaria without interfering with the preceding liver-stages of malaria parasites [12, 13]. The *P. chabaudi* model shares several characteristics with *P. falciparum*, which causes about 99% of global malaria-related deaths in humans [1].

The *P. chabaudi* model also appears appropriate to study the uncomprehended mechanisms of host defense that occur in the liver during vaccination-induced survival in blood-stage malaria. First, an effective procedure of blood-stage vaccination for *P. chabaudi* is available [14]. The non-infectious vaccine consists of erythrocyte plasma membranes isolated from *P. chabaudi*-infected erythrocytes, which contain auto-antigens and parasite-synthesized neo-antigens ([15, 16]; cf. also [17]). Immunization with this vaccine results in survival of more than 80% mice, which would have otherwise succumbed to lethal malaria by *P. chabaudi* [14, 18]. This vaccination induces a healing course of the infection and reduces peak parasitaemia by approximately 30% on day 8 post-infection (pi) and generation of long-lasting resistance against homologous re-infections [10]. Secondly, the liver of mice has been shown to respond to protective vaccination evidenced, for instance, as alterations in gene expression, miRNA expression, and DNA methylation of gene promoters upon blood-stage infection [10, 18–20].

A critical phase of *P. chabaudi* blood-stage infections is the mid-precrisis on day 4 pi, when parasitized erythrocytes begin to appear in peripheral blood. At this early patency, parasitaemia ranges between 1 and 5% varying with mice and does not differ between healing and lethal infections in vaccinated and non-vaccinated mice, respectively [18]. Moreover, early patency is associated with a dramatic decline in malaria-induced expression of multifunctional cytokines, such as IFNγ, TNF, IL-1β, and IL-6 in the liver, which drive various programmes of host defense [10, 18, 21]. Although still unexplainable at present, this decline suggests the occurrence of yet unknown processes in the liver that may be critical for vaccine efficacy and, thus, for the final outcome of blood-stage malaria. To track these processes in the liver during mid-precrisis and the possible effects by vaccination, a reasonable initial approach is to analyse global gene expression profiles in the liver for malaria-responsive genes at early patent infections of *P. chabaudi* blood-stage malaria in vaccination-protected mice in comparison with non-vaccinated unprotected mice.

## Methods

### Mice

Balb/c mice bred under specified pathogen-free conditions were obtained from the central animal facility of the University of Düsseldorf. The experiments were performed only with female mice aged 10–12 weeks. Mice were housed in plastic cages and received a standard diet (Woehrlin, Bad Salzuflen, Germany) and water ad libitum.

### Protective vaccination

Vaccination was performed under identical experimental conditions as described previously [18]. Host cell plasma membranes, isolated in the form of erythrocyte ghosts from *P. chabaudi*-parasitized erythrocytes, were used as a non-infectious vaccine, which was prepared as detailed previously [14, 22, 23]. Approximately 10^6^ ghosts were suspended in 100 µl Freund’s complete adjuvant (FCA) and subcutaneously injected at week 3 and week 1 before infection with *P. chabaudi*-parasitized erythrocytes. Control mice were treated in parallel with only FCA.

### *Plasmodium chabaudi* malaria

Blood-stage infections of *P. chabaudi* were maintained in outbred mice under sterile conditions by weekly passages of infected red blood cells. A non-clonal line of *P. chabaudi* has been used [18, 20, 24]. This line resembles *Plasmodium chabaudi chabaudi* AS in terms of restriction fragment length polymorphism analysis [25] as well as sequence identity for dihydrofolate reductase and for a cysteine protease [26] with only a single nucleotide exchange [18]. As the AS clone, the line used here has self-healing potential. However, this is controlled by sex and sex hormones, respectively, genes of the H-2 complex and genes of the non-H-2 background of the infected mouse strain [27]. Challenge of Balb/c mice with 10^6^ *P. chabaudi*-infected erythrocytes, evaluation of parasitaemia, and counting of erythrocytes were performed as described previously [18, 28]. Besides the sacrificed mice on day 0 pi and on day 4 pi, both the vaccinated group and the non-vaccinated group contained 4 ‘control’ mice, which were not sacrificed. In the non-vaccinated group, all four mice succumbed to infection during crisis, whereas only one mouse succumbed to infection during crisis in the vaccinated group, but three mice survived the infection for at least 3 weeks, in accordance with our previous results [18].

### RNA isolation

Livers were aseptically removed from sacrificed mice, rapidly frozen in liquid nitrogen, and stored at − 80 °C until use. For isolation of total RNA, livers were individually ground in a mortar under liquid nitrogen and aliquots were subjected to standard RNA extraction using Trizol. An additional RNA clean-up was followed using the miRNeasy Kit (Qiagen, Hilden, Germany). RNA integrity and quality was checked on the Agilent 2100 Bioanalyzer platform (Agilent Technologies). The RIN values of all RNA samples ranged between 8.7 and 9.1.

### RNA labelling

Each RNA sample was used to produce Cy3-labeled cRNA. Equivalents of 100 ng from individual RNA samples were amplified and labelled using the Agilent Low Input Quick Amp Labelling Kit (Agilent Technologies) according to the manufacturer’s instructions. Yields of cRNA and dye-incorporation were determined using the ND-1000 Spectrophotometer (NanoDrop Technologies). The incorporations were between 18 and 23 fmol Cy3/ng cRNA.

### Hybridization of gene expression oligo microarrays

Agilent mouse whole genome 8 × 60 K gene expression oligo microarrays (design 028005) were used for hybridization. Each microarray displayed 39,430 Entrez Gene RNAs and 16,251 long intergenic non-coding (linc)RNAs. The Agilent Gene Expression Hybridization Kit was used for hybridization as detailed in the Agilent processing protocol (Agilent technologies). In brief, 600 ng of Cy3-labelled fragmented cRNA in hybridization buffer was hybridized to the microarrays overnight at 65 °C using the recommended hybridization chamber and oven. Finally, the microarrays were washed with the Agilent Gene Expression Wash Buffer 1 (1 min at 23 °C) and then with preheated Agilent Gene Expression Wash Buffer 2 (1 min at 37 °C).

### Scanning and analyses of microarrays

Agilent microarray scanner system (Agilent Technologies) was used for detecting fluorescence signals on the hybridized microarrays. The microarray image files were processed with the Agilent feature extraction software (FES), which determines feature intensities including background subtraction, rejects outliers, and calculates statistical confidences. Three different biological replicates were performed for each sample type, i.e., 12 microarrays for four samples *in toto*. The expression variance was stabilized through the log_2_ transform. Microarrays were normalized by the quantile method. The heat map of the most highly variable transcripts, the hierarchical clustering dendrograms (calculated using the unweighted pair group method with arithmetic mean and Euclidean distance measure), and the Principal component analysis were performed using in-home functions developed in Matlab (MathWorks). The microarray data have been deposited at both the EMBL-EBI Array Express repository (Array accession number: E-MTAB.6494) and the NCBI’s Gene Expression Omnibus (GEO) database with accession number GSE111110 (https://www.ncbi.nlm.nih.gov/geo/query/acc.cgi?acc=GSE111110).

### Quantitative real-time PCR

Quantitative real-time PCR was performed under experimental conditions identical to those described recently [28], using High Capacity cDNA Reverse Transcription Kit (Life Technologies) and TaqMan mRNA assays (Life Technologies) for reverse transcription of mRNAs encoding the following proteins: ERMAP (assay ID: Mm00469273_m1), CLDN13 (Mm00491038_s1), CD163 (Mm00474091_m1), GZMB (Mm00442837_m1), KLRB1A (Mm00726548_s1), KLRC3 (Mm00650941_m1), KLRD1 (Mm00495182_m1), NCR1 (Mm01337324_g1), KLRG1 (Mm00516879_m1), and GAPDH (Mm99999915_g1). Fold change of expression was calculated using the comparative Ct method (2^−ΔΔct^) [29] and data sets were analysed for statistical significance using a two-tailed unpaired heteroscedastic Student’s t test (*p < 0.01).

## Results

### Identification of malaria-inducible genes in the liver of vaccinated and non-vaccinated mice

**Fig. 1:**
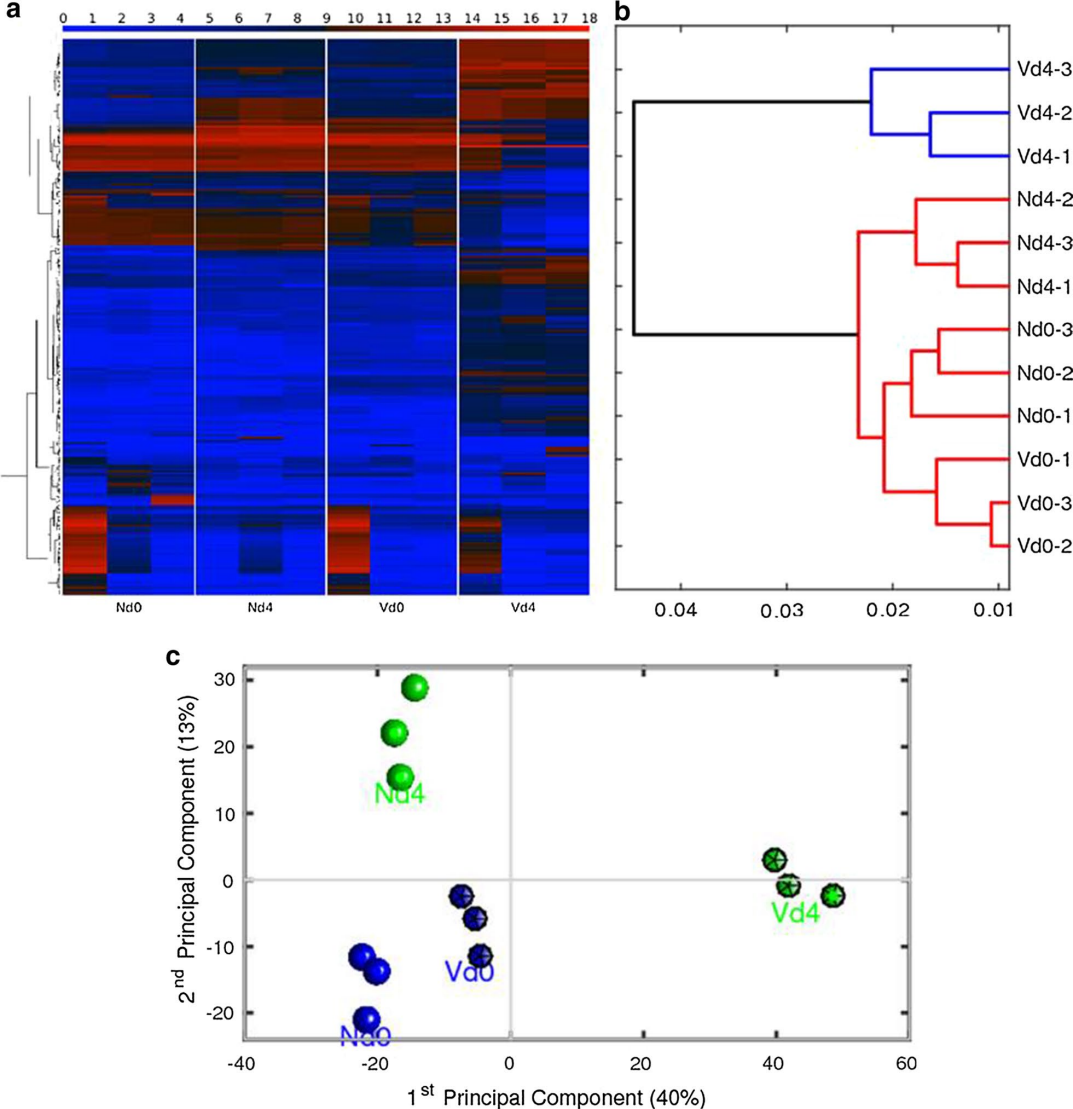
Transcriptomic global analysis of the liver from vaccination-protected Balb/c mice before infection with *Plasmodium chabaudi* on day 0 pi (Vd0 group) and after infection on day 4 pi (Vd4 group) as well as in non-vaccinated non-protected mice in the Nd0 and Nd4 groups, respectively. **a** Heatmap. The colour bar at the top codifies the gene expression in the log2 scale. Higher RNA expression corresponds to increased intensity of the colour red. **b** Hierarchical clustering of samples. **c** PCA of RNA expression data. The PC1 captures 40% of the RNA expression variability and the PC2 captures 13% of the variability

To identify malaria-induced vaccination-responsive genes in the liver at early patency, vaccinated and non-vaccinated Balb/c mice were concomitantly infected with *P. chabaudi*, and livers prepared from three vaccinated mice on day 4 pi (Vd4 group) were individually analysed with Agilent’s 8 × 60 K oligo microarrays for global gene expression in relation to those of three non-infected vaccinated mice on day 0 pi (Vd0 group). Corresponding analyses were performed with livers prepared from three non-vaccinated mice on day 4 pi (Nd4 group) in relation to those of three non-vaccinated mice on day 0 pi (Nd0 group). Figure 1a shows the global expression analysis heatmap of the most highly variable RNA expression profiles of the four different groups, each with three replicates. A group of transcripts is specifically expressed at intermediate level I in the Vd4 group, which is not expressed in the Vd0, Nd0 and Nd4 groups. Figure 1b shows the hierarchical clustering (dendrogram) of the different samples performed using the correlation metric and the average linkage method. The dendrogram reveals a clean cluster of all the three replicates in the Vd4 group and another major cluster of the data from the Nd0, Vd0, and Nd4 group, with the subclusters of samples in the Vd0 and Nd0 groups clustering closer. Principal component analysis (PCA) of the gene expression data is shown in Fig. 1c, where the 1st principal component (PC1) captures 40% of the gene expression variability and the 2nd principal component (PC2) captures 13% of the variability. The PCA indicates that the replicates of case cluster together, and that PC1, since capturing the higher percentage of gene expression variability, clearly separates all the replicates of the Nd0, Nd4, Vd0 groups from those of the Vd4 group. Taken together, these results indicate that protective vaccination does not essentially affect constitutive RNA expression of livers on day 0 pi, whereas early patent infections with *P. chabaudi* blood-stage malaria induce changes in hepatic RNA expression, differing between the Vd4 and the Nd4 group.

**Fig. 2:**
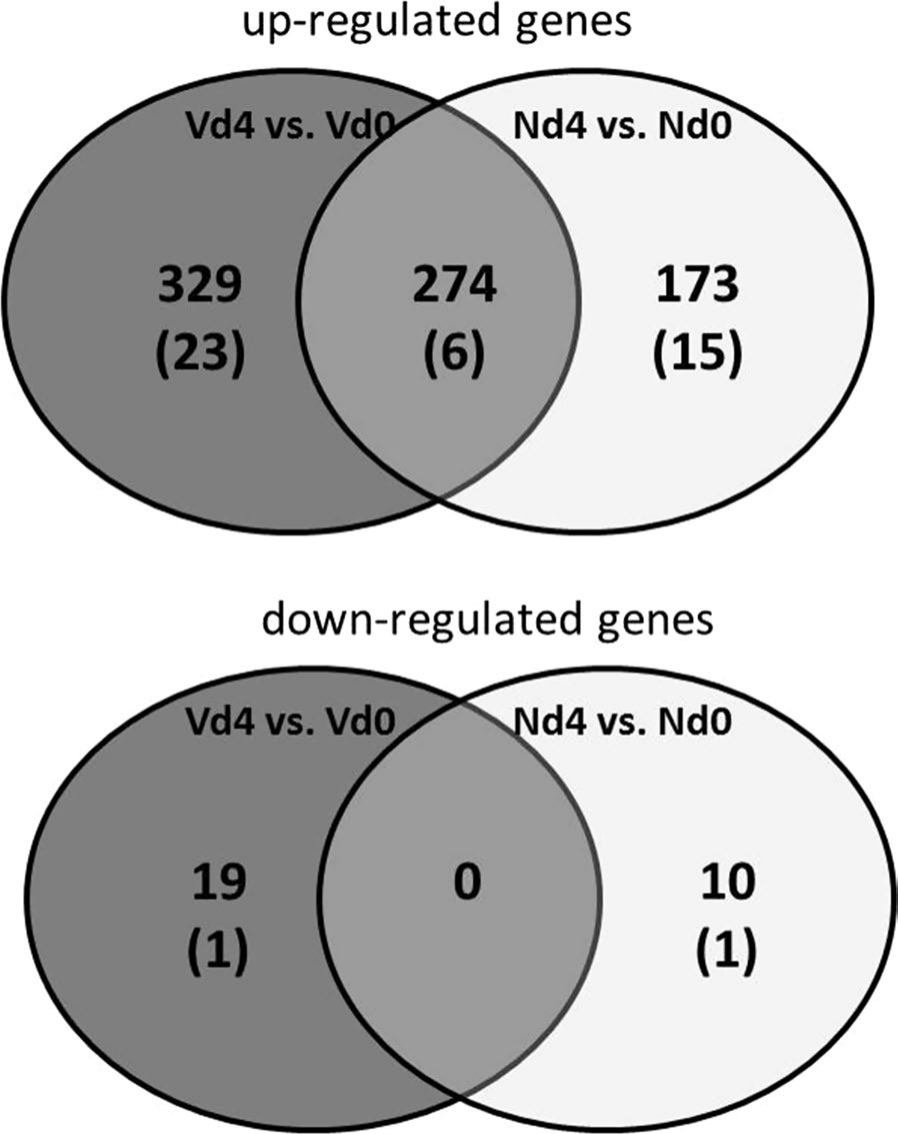
Number of genes expressed more than threefold (p < 0.01) in the liver of vaccinated mice infected with *Plasmodium chabaudi* on day 4 pi (Vd4 group) in relation to the corresponding constitutive expressions day 0 *pi* (Vd0 group). Nd4 group vs. Nd0 group indicates the number of significantly expressed genes in the liver of non-vaccinated mice. Numbers in brackets: genes with > tenfold changed expression

Malaria-induced changes in gene expression were then found in livers of both the Vd4 and Nd4 groups, when mRNAs were evaluated, which displayed > threefold changed expressions at a stringent level of significance (p < 0.01) at both d4 groups in relation to corresponding constitutive expressions of the Vd0 and Nd0 group, respectively. The total numbers of genes with significantly changed expression are summarized in the Venn diagrams shown in Fig. 2. Early patent infections induced upregulation of 329 genes in the Vd4 group, but only 173 genes in the Nd4 group, and 274 genes in both d4 groups. A remarkably lower number of genes was found to be downregulated, i.e., only 19 in the Vd4 group and still fewer, namely 10 in the Nd4 group. The Additional file 1: Tables S1 and S2 summarize the genes whose expression is up- and downregulated by > threefold and < tenfold in the Nd4 group with p < 0.01, respectively. Additional file 1: Table S3 shows 16 genes whose expression is changed by > tenfold in the Nd4 group with 15 upregulated genes and only one downregulated gene. In the Vd4 group, 306 genes were identified to be upregulated (Additional file 1: Table S4) and 18 genes were downregulated by > threefold and < tenfold, respectively (Additional file 1: Table S5). Additional file 1: Table S6 shows all genes that were significantly upregulated by > threefold in both Vd4 and Nd4 groups.

### Characterization of genes changed by > tenfold in the Vd4 group

To further restrict the number of candidate genes of potential importance for early patency and final survival, the analysis concentrated on genes, whose expressions were changed by > tenfold in the Vd4 group. Table 1 summarizes the 24 genes and their annotated functions, among which 23 were upregulated and one gene was downregulated. Conspicuously, one-third of the 23 upregulated genes are known to be involved in erythroid development. This group of genes includes *Ahsp* (48-fold) and *Kel* (66-fold), which encode the alpha haemoglobin stabilizing protein and the Kell blood group, respectively. Other genes in this group encode erythroid constituents such as the erythroblast membrane-associated protein *Ermap*, the Rhesus blood group-associated A glycoprotein, *Rhag*, and the soluble carrier family 4 *Slc4a1*, which is also termed band 3 and is one of the major erythroid integral multi-pass surface membrane proteins. The *Slc4a1*-encoded band 3 is known to function as a chloride/bicarbonate exchanger transporting carbon dioxide and to associate with glycophorin A (GYPA), which is another major integral one-pass red cell membrane protein. Remarkably, the expression of *Gypa* gene is almost tenfold higher in vaccination-protected mice (Additional file 1: Table S4), similar to *Car13* encoding carbonate anhydrase (besides *Car2* and *Car9*), which is known to interconvert carbon dioxide and bicarbonate to maintain the acid–base balance in blood and to help transport carbon dioxide out of tissues. Moreover, the group of genes upregulated by > tenfold also lists the transcription factors *Gata1* and *Gfi1b* as well as *Cldn13* upregulated by even > 43-fold.

**Table 1:** Genes expressed more or less than tenfold (p < 0.01) in the liver of vaccinated mice infected with *P. chabaudi* on day 4 p.i. (Vd4) in comparison to constitutive expression on day 0 p.i. (Vd0)

Gene	Gene description	RefSeq ID	Vd4 vs. Vd0	*p* value	Function (annotated according to www.genecards.org)
Erythropoiesis					
*Ahsp*	Alpha hemoglobin stabilizing protein	NM_133245	48.18	0.0095	Acts as a chaperone to prevent the harmful aggregation of alpha-hemoglobin during normal erythroid cell development. Specifically protects free alpha-hemoglobin from precipitation. It is predicted to modulate pathological states of alpha-hemoglobin excess such as beta-thalassemia
*Cldn13*	Claudin 13	NM_020504	43.50	0.0070	Plays a major role in tight junction-specific obliteration of the intercellular space, through calcium-independent cell-adhesion activity
*Ermap*	Erythroblast membrane-associated protein	NM_013848	21.59	0.0036	The protein encoded by this gene is a cell surface transmembrane protein that may act as an erythroid cell receptor, possibly as a mediator of cell adhesion
*Gata1*	GATA binding protein 1	NM_008089	29.73	0.0015	Transcriptional activator or repressor which probably serves as a general switch factor for erythroid development
*Gfi1b*	Growth factor independent 1B	NM_008114	20.50	0.0092	Essential proto-oncogenic transcriptional regulator necessary for development and differentiation of erythroid and megakaryocytic lineages
*Kel*	Kell blood group	NM_032540	65.89	0.0010	This gene encodes a type II transmembrane glycoprotein that is the highly polymorphic Kell blood group antigen
*Rhag*	Rhesus blood group-associated A glycoprotein	NM_011269	17.37	0.0001	The protein encoded by this gene is erythrocyte-specific and is thought to be part of a membrane channel that transports ammonium and carbon dioxide across the blood cell membrane
*Slc4a1*	Solute carrier family 4	NM_011403	11.74	0.0067	Major integral membrane glycoprotein of the erythrocyte membrane; required for normal flexibility and stability of the erythrocyte membrane and for normal erythrocyte shape via the interactions of its cytoplasmic domain with cytoskeletal proteins, glycolytic enzymes, and hemoglobin
Cell cycle and mitosis					
*Ccnb1*	Cyclin B1	NM_172301	17.76	< 0.0001	The protein encoded by this gene is a regulatory protein involved in mitosis. The gene product complexes with p34(cdc2) to form the maturation-promoting factor (MPF)
*Cdc25c*	Cell division cycle 25C	NM_009860	12.41	0.0009	Cdc25 activates cdk complexes that drive the cell cycle. Cdc25 is involved in the DNA damage checkpoints and is known as a key mediator of cell cycle progression
*Ckap2* l	Cytoskeleton associated protein 2-like	NM_181589	10.72	0.0030	Microtubule-associated protein required for mitotic spindle formation and cell-cycle progression in neural progenitor cells
Innate immunity					
*Abcg4*	ATP-binding cassette, sub-family G (WHITE), member 4	NM_138955	26.79	0.0035	The protein encoded by this gene is included in the superfamily of ATP-binding cassette (ABC) transporters. May be involved in macrophage lipid homeostasis
*Ccl7*	Chemokine (C-C motif) ligand 7	NM_013654	34.97	0.0013	Chemotactic factor that attracts monocytes and eosinophils, but not neutrophils
Socs1	Suppressor of cytokine signaling 1	NM_009896	11.32	0.0070	This gene encodes a member of the STAT-induced STAT inhibitor (SSI), also known as suppressor of cytokine signaling (SOCS), family. SSI family members are cytokine-inducible negative regulators of cytokine signaling. The expression of this gene can be induced by a subset of cytokines, including IL2, IL3 erythropoietin (EPO), CSF2/GM-CSF, and interferon (IFN)-gamma
*Treml2*	Triggering receptor expressed on myeloid cells-like 2	NM_001033405	10.92	0.0001	Cell surface receptor that may play a role in the innate and adaptive immune response. Acts as a counter-receptor for CD276 and interaction with CD276 on T-cells enhances T-cell activation
*Cd163*	CD163 antigen	NM_053094	0.03	0.0018	The protein encoded by this gene is a member of the scavenger receptor cysteine-rich (SRCR) superfamily, and is exclusively expressed in monocytes and macrophages. It functions as an acute phase-regulated receptor involved in the clearance and endocytosis of hemoglobin/haptoglobin complexes by macrophages, and may thereby protect tissues from free hemoglobin-mediated oxidative damage
Cytotoxicity					
*Gzmb*	Granzyme B	NM_013542	35.66	0.0085	The encoded preproprotein is secreted by natural killer (NK) cells and cytotoxic T lymphocytes (CTLs) and proteolytically processed to generate the active protease, which induces target cell apoptosis. This protein also processes cytokines and degrades extracellular matrix proteins, and these roles are implicated in chronic inflammation and wound healing
*Klrb1a*	Killer cell lectin-like receptor subfamily B member 1A	NM_010737	10.43	0.0083	Plays an inhibitory role on natural killer (NK) cells cytotoxicity. Activation results in specific acid sphingomyelinase/SMPD1 stimulation with subsequent marked elevation of intracellular ceramide. Activation also leads to AKT1/PKB and RPS6KA1/RSK1 kinases stimulation as well as markedly enhanced T-cell proliferation induced by anti-CD3
*Klrc3*	Killer cell lectin-like receptor subfamily C, member 3	NM_021378	11.38	0.0002	KLRC3 is a member of the NKG2 group which are expressed primarily in natural killer (NK) cells and encodes a family of transmembrane proteins characterized by a type II membrane orientation (extracellular C terminus) and the presence of a C-type lectin domain
*Klrd1*	Killer cell lectin-like receptor, subfamily D, member 1	NM_010654	16.33	0.0067	Several genes of the C-type lectin superfamily, including members of the NKG2 family, are expressed by NK cells and may be involved in the regulation of NK cell function. Plays a role as a receptor for the recognition of MHC class I HLA-E molecules by NK cells and some cytotoxic T-cells
*Ncr1*	Natural cytotoxicity triggering receptor 1	NM_010746	16.97	0.0084	Cytotoxicity-activating receptor that may contribute to the increased efficiency of activated natural killer (NK) cells to mediate tumor cell lysis
Miscellaneous					
*Hist1h3g*	Histone cluster 1, H3g	NM_145073	18.90	0.0075	Core component of nucleosome
*Htr7*	5-Hydroxytryptamine (serotonin) receptor 7	NM_008315	12.53	< 0.0001	Serotonin 5-HT7 receptors are located primarily in the thalamus, hypothalamus and hippocampus. The function of these receptors includes the regulation of circadian rhythms, thermoregulation, learning and memory and smooth muscle relaxation
*Nxpe5*	Neurexophilin and PC-esterase domain family, member 5	NM_001013773	22.61	0.0089	Unknown

Furthermore, a group of five genes encoding proteins, known to be involved in cellular cytotoxicity, such as *Gzmb* encoding granzyme B (36-fold), *Ncr1* encoding the natural cytotoxicity triggering receptor 1, and *Klrb1a*, *Klrc3*, and *Klrd1* encoding three different killer cell lectin-like receptors, were upregulated by > tenfold (Table 1). Remarkably, among the six genes significantly upregulated by > tenfold in the liver in both Vd4 and Nd4 groups (Additional file 1: Table S7), one gene, *Klrg1*, encoding the killer cell lectin-like receptor subfamily G, was overexpressed by at least 100% in the Vd4 group compared to the Nd4 group, i.e., 39.5-fold vs. 14.4-fold.

Another group of genes in Table 1 includes the 3 genes *Ccbn1*, *Cdc25c*, and *Ckap 21* that were upregulated between 10- and 18-fold, and are known to be involved in cell cycle control including mitosis (Table 1). Table 2 also shows 22 genes extracted from Additional file 1: Table S6, whose expression is significantly upregulated by > tenfold in the Vd4 group and, concomitantly, by at least 100% more than the corresponding genes significantly expressed in the Nd4 group. Conspicuously, 15 of these 22 genes are apparently involved in mitosis, particularly in the formation and function of the mitotic spindle. The two genes *Prr11* and *Sapcd2* are critically involved in cell cycle progression. The gene *Klrb1f* encodes a killer cell lectin-like receptor, which plays an inhibitory role in NK cell cytotoxicity. *Birc5* is still remarkable because it codes for a negative regulatory protein preventing apoptotic cell death.

**Table 2:** Hepatic expression of genes up-regulated by more than tenfold (p < 0.01) at Vd4 and by 100% more than at Nd4

Gene	Gene description	RefSeq ID	Vd4 vs. Vd0	*p* value	Nd4 vs. Nd0	*p* value	Function (annotated according to www.genecards.org)
Mitosis							
*Bub1*	Budding uninhibited by benzimidazoles 1 homolog	NM_009772	20.11	0.0049	5.88	0.0021	Serine/threonine-protein kinase that performs two crucial functions during mitosis: it is essential for spindle-assembly checkpoint signaling and for correct chromosome alignment
*Espl1*	Extra spindle poles-like 1	NM_001014976	12.35	0.0013	4.98	0.0020	Caspase-like protease, which plays a central role in the chromosome segregation by cleaving the SCC1/RAD21 subunit of the cohesin complex at the onset of anaphase
*Mki67*	Antigen identified by monoclonal antibody Ki 67	NM_001081117	13.08	0.0004	4.29	0.0014	Required to maintain individual mitotic chromosomes dispersed in the cytoplasm following nuclear envelope disassembly
*Mxd3*	Max dimerization protein 3	NM_016662	10.52	0.0031	3.84	0.0066	This gene encodes a member of the Myc superfamily of basic helix-loop-helix leucine zipper transcriptional regulators. The encoded protein forms a heterodimer with the cofactor MAX which binds specific E-box DNA motifs in the promoters of target genes and regulates their transcription
*Ndc80*	NDC80 homolog, kinetochore complex component	NM_023294	11.19	0.0004	4.74	0.0005	Acts as a component of the essential kinetochore-associated NDC80 complex, which is required for chromosome segregation and spindle checkpoint activity
*Nusap1*	Nucleolar and spindle associated protein 1	NM_133851	14.19	0.0002	4.89	0.0005	NUSAP1 is a nucleolar-spindle-associated protein that plays a role in spindle microtubule organization
*Plk1*	Polo-like kinase 1	NM_011121	14.14	0.0035	4.13	0.0018	Serine/threonine-protein kinase that performs several important functions throughout M phase of the cell cycle, including the regulation of centrosome maturation and spindle assembly, the removal of cohesins from chromosome arms, the inactivation of anaphase-promoting complex/cyclosome (APC/C) inhibitors, and the regulation of mitotic exit and cytokinesis
*Prc1*	Protein regulator of cytokinesis 1	NM_145150	15.38	< 0.0001	4.28	0.0003	The protein is present at high levels during the S and G2/M phases of mitosis but its levels drop dramatically when the cell exits mitosis and enters the G1 phase
*Racgap1*	Rac GTPase-activating protein 1	NM_001253809	12.67	0.0042	3.83	0.0003	Component of the centralspindlin complex that serves as a microtubule-dependent and Rho-mediated signaling required for the myosin contractile ring formation during the cell cycle cytokinesis
*Ska1*	Spindle and kinetochore associated complex subunit 1	NM_025581	23.19	0.0004	5.46	0.0039	Component of the SKA1 complex, a microtubule-binding subcomplex of the outer kinetochore that is essential for proper chromosome segregation
*Ska3*	Spindle and kinetochore associated complex subunit 3	NM_198605	15.24	0.0027	4.68	0.0013	This gene encodes a component of the spindle and kinetochore-associated protein complex that regulates microtubule attachment to the kinetochores during mitosis
*Ticrr*	TOPBP1-interacting checkpoint and replication regulator	NM_029835	14.22	0.0008	4.66	0.0093	Regulator of DNA replication and S/M and G2/M checkpoints. Regulates the triggering of DNA replication initiation via its interaction with TOPBP1 by participating in CDK2-mediated loading of CDC45L onto replication origins
*Tpx2*	TPX2, microtubule-associated protein homolog	NM_028109	16.11	0.0032	4.28	0.0011	Spindle assembly factor required for normal assembly of mitotic spindles. Required for normal assembly of microtubules during apoptosis
*Ube2c*	Ubiquitin-conjugating enzyme E2C	NM_026785	12.49	< 0.0001	4.32	0.0010	Essential factor of the anaphase promoting complex/cyclosome (APC/C), a cell cycle-regulated ubiquitin ligase that controls progression through mitosis
Cell cycle/cell signaling							
*Klrb1f*	Killer cell lectin-like receptor subfamily B member 1F	NM_153094	10.63	0.0005	5.32	0.0003	Plays an inhibitory role on natural killer (NK) cells cytotoxicity. Activation results in specific acid sphingomyelinase/SMPD1 stimulation with subsequent marked elevation of intracellular ceramide
*Birc5*	Baculoviral IAP repeat-containing 5	NM_009689	16.68	0.0005	3.47	0.0006	This gene is a member of the inhibitor of apoptosis (IAP) gene family, which encode negative regulatory proteins that prevent apoptotic cell death
*Cd7*	CD7 antigen	NM_009854	13.52	0.0022	4.54	0.0005	Plays an essential role in T-cell interactions and also in T-cell/B-cell interaction during early lymphoid development
*Kif11*	Kinesin family member 11	NM_010615	11.80	0.0065	3.62	0.0077	Motor protein required for establishing a bipolar spindle during mitosis
*Prr11*	Proline rich 11	NM_175563	12.13	0.0001	4.52	0.0001	Plays a critical role in cell cycle progression
*Sapcd2*	Suppressor APC domain containing 2	NM_001081085	11.96	0.0001	3.30	0.0040	Plays a critical role in cell cycle progression
Miscellaneous							
*Iqgap3*	IQ motif containing GTPase activating protein 3	NM_001033484	16.36	0.0002	3.88	0.0019	Unknown
*Raet1c*	Retinoic acid early transcript gamma	NM_009018	15.69	0.0001	4.83	0.0011	Unknown

**Fig. 3:**
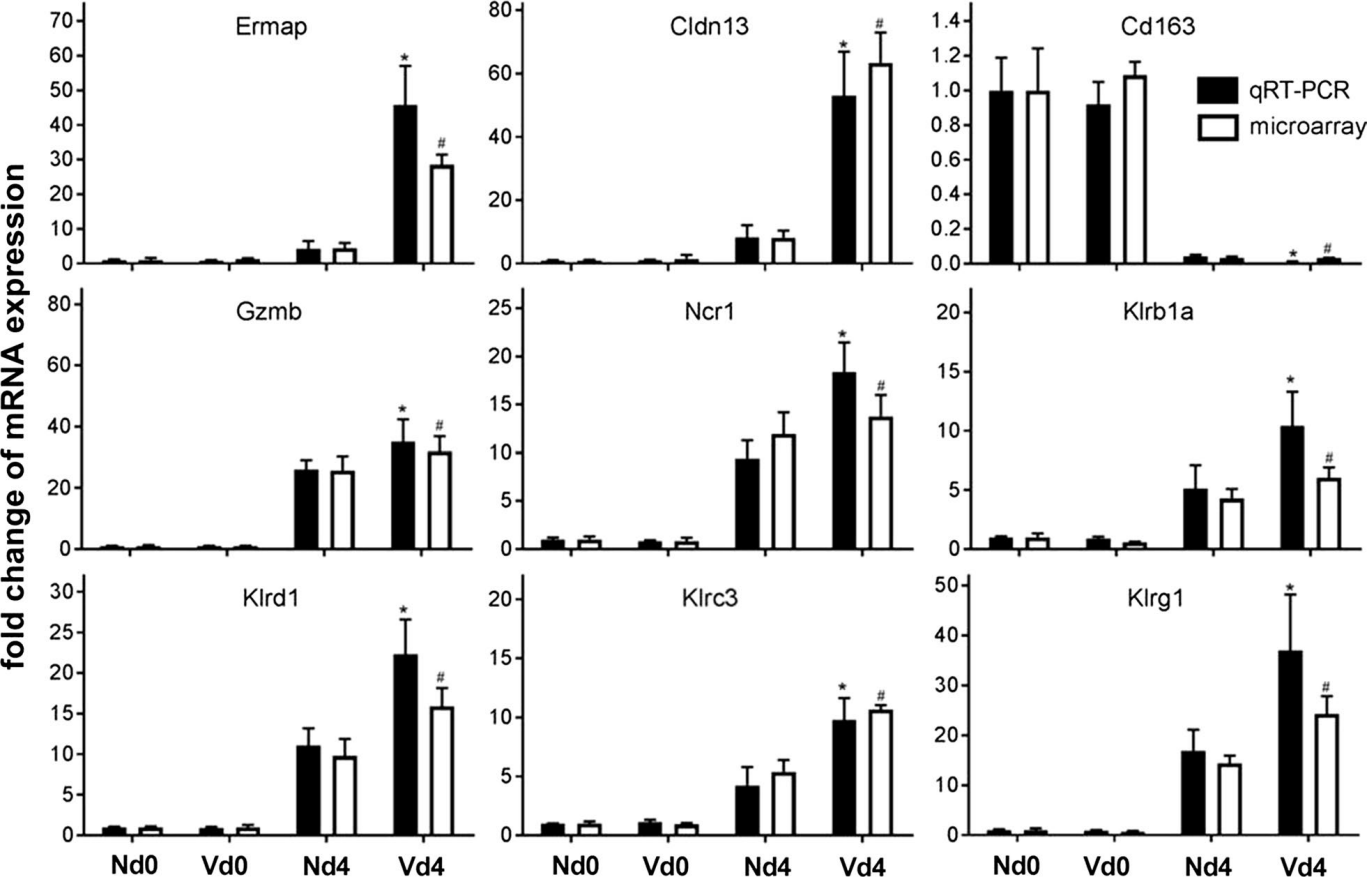
Quantitative PCR of mRNAs in the liver of Balb/c mice in comparison with the microarray data. Livers were isolated from three vaccinated mice before infection with *Plasmodium chabaudi* on day 0 pi (Vd0 group), from three vaccinated mice after infection on day 4 pi (Vd4 group), and from three non-vaccinated mice at Nd0 group and at Nd4 group. Means of duplicate determinations, performed with liver aliquots from three different mice, with half SEM. Stars and hashtags indicate significant differences (p < 0.01) between Vd4 and Vd0 groups as revealed by qRT-PCR and microarrays, respectively

Among the four genes involved in innate immunity, expression of the suppressor of cytokine signaling 1, *Socs1*, was upregulated approximately by 11-fold (Table 1). *Cd163* is the only gene whose expression was significantly downregulated by > tenfold in the Vd4 group (Table 1; Fig. 3).

Finally, quantitative PCR was used to reexamine the expression of some of the genes, which were identified to be expressed by > tenfold in the Vd4 group in the microarrays, particularly *Ermap*, *Cldn13*, *Cd163*, *Gzmb*, *Ncr1*, *Klrb1a*, *Klrd1*, *Klrc3*, and *Klrg1*. Figure 3 shows that the constitutive expression of these genes was not significantly affected by protective vaccination. However, early patent infections of *P. chabaudi* significantly changed the expression of these genes in the Vd4 group, which is comparable with the result of the microarrays.

### LincRNAs expressed in vaccination-protected mice

**Fig. 4:**
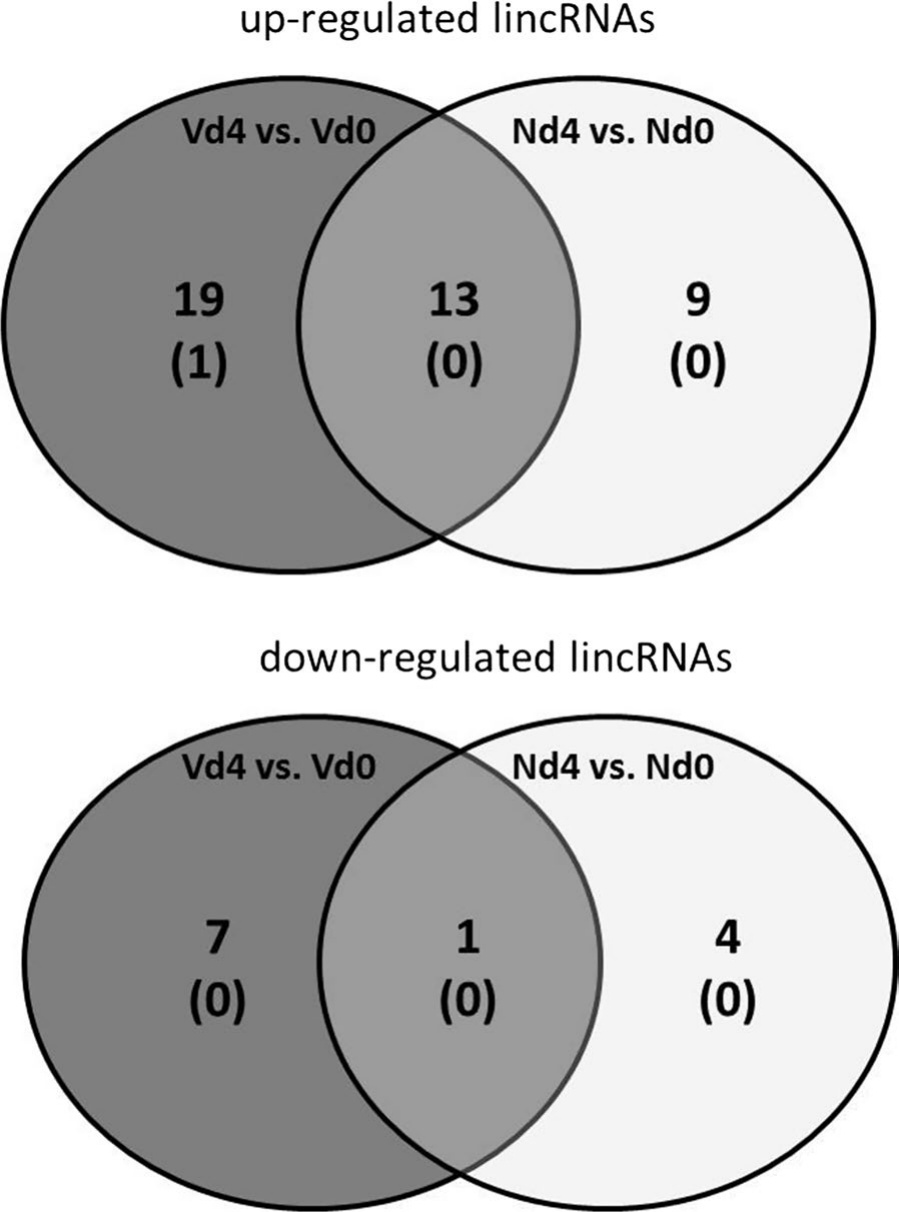
Number of lincRNAs with expressions changed by > threefold (p < 0.01) in the liver of non-vaccinated (N) and vaccinated mice (V) infected with *Plasmodium chabaudi* on day 4 pi in relation to their constitutive expression in the Nd0 and Vd0 groups, respectively. Numbers in brackets indicate the lincRNAs with > tenfold changed expressions

There is increasing evidence that long non-coding (lnc)RNAs including long intergenic non-coding (linc)RNAs are critical for the course and outcome of different diseases, including diverse liver diseases [30]. These non-coding RNAs range in size between 200 bp and ~ 100 kb. LincRNAs do not overlap with annotated coding regions *per definitionem*, though an increasing number of lincRNAs has been recently identified to contain small open reading frames coding for small functional peptides [31]. Moreover, lincRNAs are widely transcribed in mammalian cells, though at lower levels and in a more cell-type specific manner than mRNA [32–34]. The used microarrays contain 16,251 lincRNA probes and have been evaluated for lincRNA expression using the same stringent conditions as those used for mRNAs. Early patent infections of *P. chabaudi* blood-stage malaria induce changes in the expression of lincRNAs, which differ significantly (p < 0.01) between vaccination-protected and non-protected non-vaccinated mice (Fig. 4). In vaccination-protected mice, 7 lincRNAs are downregulated and 19 lincRNAs are upregulated by > threefold (Additional file 1: Table S8), among which one lincRNA is upregulated by > tenfold. In addition, 13 lincRNAs are upregulated in the Vd4 group in common with the Nd4 group and 1 lincRNA is downregulated (Additional file 1: Table S9).

Remarkably, the two lincRNA species lincRNA:chr10:117021051-117038683 forward strand and lincRNA:chr2:84344350-843770075 reverse strand are more highly expressed in the Vd4 group (by > 100%) than in the Nd4 group. LincRNA species that are significantly up- and down-regulated in non-vaccinated mice in the Nd4 group (Additional file 1: Table S9) differ from those identified in vaccination-protected mice in the Vd4 group. Annotated functions are not yet available for any of these differentially regulated lincRNAs.

## Discussion

This study provides evidence that the hepatic response in terms of mRNA and lincRNA expression, to *P. chabaudi* blood-stage malaria at early patency differs between vaccination-induced healing infections and lethal infections in non-vaccinated mice. In particular, 24 genes are altered by > tenfold at p < 0.01 in the liver of vaccination-protected mice. In addition, there are 22 genes > tenfold expressed in vaccination-protected mice which are at least 100% higher induced than the corresponding genes significantly expressed in non-vaccinated mice. These data indicate that, at early patency, critical processes occur in the liver, which may contribute to vaccination-induced survival of blood-stage infections.

One of these processes may be extramedullary erythropoiesis in the liver. Indeed, approximately one-third of the 23 genes induced in vaccination-protected mice by > tenfold are erythroid-associated genes, encoding the Kell blood group, the Rhesus blood group-associated glycoprotein A, extrinsic and intrinsic membrane proteins such as ERMAP, SLC4A1 (band 3), and glycophorin A, as well as the transcription factors GATA1 and GFI1b, which are key regulators of erythroid development [28]. These genes were not identified to be significantly (p < 0.01) expressed in non-vaccinated mice, except for *Ermap*, which was induced only by about fivefold in the Nd4 group (Additional file 1: Table S1).

Extramedullary erythropoiesis in the liver of vaccination-protected mice has been recently shown to occur towards the end of the crisis phase on day 11 pi [28]. Crisis is characterized by much higher expression of erythroid-associated genes than that described here at early patency on day 4 pi For instance, the genes *Ermap*, *Slca1*, *Gata1*, and *Gfi1b* are expressed by > 100-fold at crisis. As previously shown under identical experimental conditions, crisis in vaccination-protected mice is also characterized by a dramatic decrease in peripheral *P. chabaudi*-parasitized erythrocytes and, concomitantly, in a dramatic increase in the number of peripheral reticulocytes, the latter being impaired in non-vaccinated mice [18].

Incidentally, reticulocytes are not the preferred host cells for *P. chabaudi* [35]. At early patency, however, *P. chabaudi*-parasitized erythrocytes only begin to appear in the peripheral blood [18]. The number of peripheral reticulocytes is still very low and not yet essentially changed [18]. These data suggest: (1) that extramedullary erythropoiesis occurring in the liver at early patency of the malaria blood-stage infections is still in an early state: and, (2) that extramedullary erythropoiesis in the liver is apparently accelerated in vaccination-protected mice in comparison to non-vaccinated mice.

There is evidence indicating that stress, including psychological stress, chemicals, and diverse viral and bacterial infections, can induce extramedullary erythropoiesis in several organs, particularly in the spleen, of mice and even humans [36–46]. Even endo- and ecto-parasites such as ticks [47] or *Trypanosoma congolense* [48] are able to induce extramedullary erythropoiesis in the spleen of their hosts. Remarkably, the latter has been found to be associated with increased expression of the apparent mouse-specific gene, *Cldn13*, encoding the most abundant claudin of the 26-membered claudin family in the spleen [49, 50]. Claudins are the main constituents of tight junctions; however, CLDN13 has been predicted to be localized on the surface of erythroblasts in the spleen [48]. Previously, a massively upregulated expression of *Cldn13* by > 100-fold has been found towards the end of the crisis phase of *P. chabaudi* blood-stage infections in vaccination-protected mice and it was therefore suggested that *Cldn13* is locally expressed in/around erythroblast islands in the liver [28]. The present data would then indicate that *Cldn13* is already expressed at early extramedullary erythropoiesis in the liver of vaccination-protected mice. Another gene possibly involved in liver erythropoiesis may be *Cd163*, which is the only gene found to be downregulated by > tenfold, since the encoded transmembrane scavenger receptor CD163 on the surface of Kupffer cells has been described to serve not only in clearance and endocytosis of haemoglobin/haptoglobin complexes [51–53], but also as an adhesion factor for erythroblasts in erythroblastic islands [53, 54].

An increase in killer cells, i.e., NK cells, NKT cells, and cytolytic CD8^+^ cells [55], may also occur in the liver of vaccination-induced healing infections at early patency. This view is supported by the present finding that the granzyme b gene *Gzmb*, the natural cytotoxicity-triggering receptor 1 gene *Ncr1*, and the killer cell lectin–lectin like receptor genes *Klrb1a*, *Klrc3*, *Klrd1*, and *Klrg1* are massively upregulated by > tenfold in vaccination-protected mice. KLRs, GZMB inducing apoptosis in target cells via the caspase-mediated apoptotic pathway, and NCR1 are known to be predominantly expressed on NK cells, though NCR1 is also expressed on type 1 innate lymphoid cells [56–59]. The increased mRNA levels encoded by *Klrs*, *Gzmb*, *and Ncr1* might be interpreted as to be due to an immigration of peripheral c(conventional)NK cells [55] from circulation into the liver. On the other hand, however, the major lymphocyte population in the liver is presumably another subset of NK cells, namely liver-resident NK cells developing from progenitor cells in the liver [55]. Increasing evidence indicates that the liver-resident NK cell subset differs in phenotype and function from cNK cells [55, 60], though both NK cell subsets produce about the same high levels of GZMB [59]. In contrast to the CD49a^−^DX5^+^cNK cells, the liver-resident NK cells are CD49a^+^DX5^−^ and even differ, also in terms of gene expression signatures, from cNK cells and other tissue-resident NK cells, as e.g. those distinct lineages of NK cells occurring in spleen, thymus, and uterus [61]. Thus, it is more attractive to speculate that the upregulated mRNA levels of the different NK cell markers found here to be induced by blood-stage malaria in the liver of vaccination-protected mice may reflect an intra-hepatic accelerated generation of liver-resident NK cells.

Liver-resident NK cells have been described to exert numerous functions, but their predominant function is killing of target cells using different apoptotic pathways [55, 60, 62]. For instance, NK cells kill myofibroblasts, which are known to induce liver fibrosis, thus limiting the spread of fibrosis in the liver [62]. There is evidence that NK cells are also able to kill *Plasmodium*-parasitized erythrocytes thus contributing to protection from murine and human malaria [63–67]. It is therefore plausible to assume that NK cells attack *P. chabaudi*-infected erythrocytes in the liver thus transforming the normally tolerogenic milieu of the liver to an increasingly hostile parasite environment, at least at early patency when *P. chabaudi*-infected erythrocytes begin to appear in the peripheral blood. Remarkably, an increased NK cell activity, in terms of the here found upregulated genes of NK cell markers, has not been previously observed towards the end of the crisis phase in vaccination-protected mice when there is a massive appearance of reticulocytes in the peripheral blood [28]; concomitantly, the liver has been shown to dramatically increase its uptake of particulate material [18] including *P. chabaudi*-parasitized erythrocytes [68]. Thus, it is possible that the increased generation of NK cells in the liver of vaccination-protected mice at early patency may not fortuitously correlate with early erythropoiesis in the liver.

Indeed, a recent report described that murine *Cytomegalovirus* (MCMV) infections induce extramedullary haematopoiesis in the spleen with a dominance of the red blood cell lineage [62]. The development of this extramedullary haematopoiesis requires the cytotoxic function of NK cells rather than their cytokine production. This cytotoxic activity of NK cells is obviously responsible for confining virus spread, thereby protecting extramedullary haematopoietic niches and facilitating extramedullary haematopoiesis, which otherwise is suppressed by MCMV [69]. Depression of cytokine signaling in the liver of vaccination-protected mice at early patency is indicated by a dramatic decline in the expression of *Ifnγ* and *Tnfα* [18]. This is predictable because the expression of *Socs1* encoding the suppressor of cytokine signaling is increased by > tenfold in the liver of vaccination-protected mice, but not in non-vaccinated mice at early patency. It is, therefore, possible that *Socs1* is critically involved in the accelerated generation of liver-resident killer cells, particularly NK cells.

Accelerated extramedullary erythropoiesis and generation of killer cells in the liver may also explain why the present study identified a group of genes known to be involved in cell cycle regulation and especially mitosis i.e., *Ccbn1*, *Cdc25c*, and *Ckap 21*, at early patency in the liver of vaccination-protected mice. Additionally, the vast majority of the > tenfold expressed 22 genes, which are at least 100% higher induced in vaccination-protected mice than the corresponding genes significantly expressed in non-vaccinated mice, is known to be involved in mitosis and cell cycle control. Even erythroblast enucleation during erythroid development can be regarded as an asymmetric mitosis [28, 48]. However, several of these genes such as *Bub1*, *Nusap1*, *Prc1*, *Ska1*, and *Ube2c*, have also previously been found to be expressed at about the same level, as here at early patency, towards the end of crisis in vaccination-protected mice [28]. Thus, it is not unlikely that the changes in the expression of these cell cycle and mitosis controlling genes reflect accelerated extramedullary erythropoiesis and generation of liver-resident cytotoxic cells and may also be associated with accelerated liver regeneration in general. Indeed, there is evidence that the liver during the acute phase of *P. chabaudi* blood-stage malaria is pathologically damaged and even heavily injured with distant effects on other organs such as in hepatoencephalopathy [9, 10]. Even *Plasmodium falciparum* and *Plasmodium vivax* malaria in humans is associated with massive liver dysfunction [70–73]. Accelerated liver regeneration may therefore contribute to accelerated recovery from the malaria-induced dysfunctions of the liver [20, 28].

Finally, the present data demonstrate that *P. chabaudi* blood-stage malaria does not only alter gene expression in the liver at early patency, but also affects the expression of lincRNAs, and this lincRNA expression has changed after protective vaccination. The identified lincRNAs are not yet functionally annotated, as it is typical for most other known lincRNAs [31]. In general, however, evidence is increasing that lncRNAs including lincRNAs play a critical role in nuclear organization and chromatin remodeling, in cell-type specific activation and repression of gene expression through diverse mechanisms, in tissue-specific fine-tuning of the expression of neighbouring genes, in regulation of cell-lineage development, and in course and outcome of diverse diseases including liver diseases [31, 33, 34, 74–76]. Here, several lincRNA species have been identified in the liver, whose malaria-induced expression is increased by protective vaccination during mid-precrisis on day 4 pi, and whose expression is still more increased in the liver of vaccination-protected mice towards the end of the crisis phase on day 11 pi as described recently [28]. For instance, expression of the lincRNA:chr12:32781477-32808567 is upregulated from 6.6 in the Vd4 group to 71.9 in the Vd11 group, the lincRNA:chr5:77084398-77086144 reverse strand from 10.7 to 19.9, the lincRNA:chr15:61984389-62102500 reverse strand from 3.2 to 6.1, and the lincRNA:chr10:83980790-83986015 reverse strand from 3.1 to 9.7, respectively. At least these 4 lincRNA species in the liver may be speculated to contribute to vaccination-induced healing of the otherwise lethal *P. chabaudi* malaria infections. Currently, the role of lncRNAs is still poorly understood with respect to extramedullary erythropoiesis and/or generation of liver-resident killer cells and/or hepatic regeneration [62] and/or megakaryopoiesis in the liver [20]. Only erythropoiesis has been recently shown to be associated with diverse lncRNAs [75, 76], particularly in steps of erythropoiesis that are targeted by the transcription factor GATA1 [77, 78]. The expression of *Gata1* in the liver was here detected to be upregulated by > tenfold at early patency during the pre-crisis phase and, still more, by > 100-fold towards the end of the crisis phase in vaccination-protected mice [28]. One study has shown [77] that when the erythroid-specific lncRNA species alncRNA-EC7, also known as Bloodlinc, is knocked down, the expression of the 10 kb away located gene *Slc4a1*, which encodes the band 3 erythrocyte membrane protein and which is found here to be expressed by more than tenfold at early patency and by more than 100-fold at crisis in the liver of vaccination-protected mice [28], is decreased by 80%. Specifically, Bloodlinc is located in the coordinates chr11:102,231,615–102,237,204 of the version mm9 of the mouse genome, which is also used for annotation of our lincRNA containing arrays; incidentally, the latter do not contain any specific probes for the lncRNA Bloodlinc.

Collectively, the present data indicate that protective vaccination changes the hepatic response in terms of mRNA and lincRNA expression, to early patent healing infections of *P. chabaudi* blood-stage malaria. These changes are suggested to be associated with an accelerated occurrence of extramedullary erythropoiesis, generation of liver-resident cytotoxic cells, and liver regeneration. These accelerated processes at early patency may be of critical importance for the final vaccination-induced healing outcome of the otherwise lethal blood-stage *P. chabaudi* malaria.

## Additional file

**Additional file 1: Table S1.** Genes, whose expression is up-regulated more than 3-fold and less than 10-fold (p < 0.01) in the liver of non-vaccinated mice infected with P. chabaudi on day 1 p.i. (Nd1) in comparison to constitutive expression on day 0 p.i. (Nd0). **Table S2.** Genes, whose expression is down-regulated more than 3-fold and less than 10-fold (p < 0.01) in the liver of non-vaccinated mice infected with P. chabaudi on day 1 p.i. (Nd1) in comparison to constitutive expression on day 0 p.i. (Nd0). **Table S3.** Genes up-regulated and down-regulated more than 10-fold (p < 0.01) in the liver of vaccinated mice infected with P. chabaudi on day 1 p.i. (Nd1) in relation to constitutive expression on day 0 p.i. (Nd0. **Table S4.** Genes, whose expression is up-regulated more than 3-fold and less than 10-fold (p < 0.01) in the liver of both non-vaccinated (N) and vaccinated mice (V) infected with P. chabaudi on day 1 p.i. (Vd1, Nd1) in comparison to constitutive expression on day 0 p.i. (Vd0, Nd0). **Table S5.** Genes, whose expression is down-regulated more than 3-fold and less than 10-fold (p < 0.01) in the liver of both non-vaccinated (N) and vaccinated mice (V) infected with P. chabaudi on day 1 p.i. (Vd1, Nd1) in comparison to constitutive expression on day 0 p.i. (Vd0, Nd0). **Table S6.** Genes expressed more than 3-fold and less than 10-fold (p < 0.01) in the liver of vaccinated mice infected with P. chabaudi on day 1 p.i. (Vd1) in comparison to constitutive expression on day 0 p.i. (Vd0). **Table S7.** Genes down-regulated more than 3-fold and less than 10-fold (p < 0.01) in the liver of vaccinated mice infected with P. chabaudi on day 1 p.i. (Vd1) in comparison to constitutive expression on day 0 p.i. (Vd0). **Table S8.** LincRNAs up-regulated more than 3-fold (p < 0.01) in the liver of vaccinated mice infected with P. chabaudi on day 1 p.i. (Vd1) in comparison to constitutive expression on day 0 p.i. (Vd0).**Table S9.** LincRNAs down-regulated more than 3-fold (p < 0.01) in the liver of vaccinated mice infected with P. chabaudi on day 1 p.i. (Vd1) in comparison to constitutive expression on day 0 p.i. (Vd0).
